# 433. Role of Community Vaccination Coverage in Controlling Future COVID-19 Outbreaks in Nursing Homes: A Modeling Study

**DOI:** 10.1093/ofid/ofab466.633

**Published:** 2021-12-04

**Authors:** Brajendra K Singh, Joseph Walker, Prabasaj Paul, Sujan Reddy, John A Jernigan, John A Jernigan, Rachel Slayton

**Affiliations:** 1 Centers for Disease Control and Prevention, Atlanta, Georgia; 2 CDC, Atlanta, Georgia

## Abstract

**Background:**

As of May 2, 2021, U.S. nursing homes (NHs) have reported >651,000 COVID-19 cases and >132,000 deaths to CDC’s National Healthcare Safety Network. Since U.S. COVID-19 vaccination coverage is increasing, we investigate the role of vaccination in controlling future COVID-19 outbreaks.

**Methods:**

We developed a stochastic, compartmental model of SARS-CoV-2 transmission in a theoretical 100-bed NH with a staff of 99 healthcare personnel (HCP) in a community of 20,000 people. We modeled admission and discharge of residents (parameterized with Centers for Medicare & Medicaid Services data), assuming the following: temporary replacement of HCP when tested positive; daily visits to NH residents; isolation of COVID-19 positive residents; personal protective equipment (PPE) use by HCP; and symptom-based testing of residents and staff plus weekly asymptomatic testing of HCP and facility-wide outbreak testing once a COVID-19 case is identified. We systematically varied coverage of an mRNA vaccine among residents and HCP, and in the community. Simulations also varied PPE adherence, defined as the percentage of time in the facility that HCP properly used recommended PPE (25%, 50% or 75% of the time). Infection was initialized in the community with 40 infectious cases, and initial infection in the NH was allowed after 14 days of vaccine dose 1. Simulations were run for 6 months after dose 2 in the NH. Results were summarized over 1000 simulations.

**Results:**

At 60% community coverage, expected cumulative symptomatic resident cases over 6 months were ≤5, due to low importation of COVID-19 infection from the community, with further reduction at higher coverage among HCP (Figure 1). Uncertainty bounds narrowed as NH resident coverage or PPE adherence increased. Results were similar if testing of staff and residents stopped. Probability of an outbreak within 4 weeks of dose 2 remained below 5% with high community coverage (Figure 2).

Figure 1. Drop in symptomatic cases in nursing home (NH) residents with rise in COVID-19 vaccine coverage in the community, increase in personal protective equipment (PPE) adherence, or increase in coverage among NH residents.

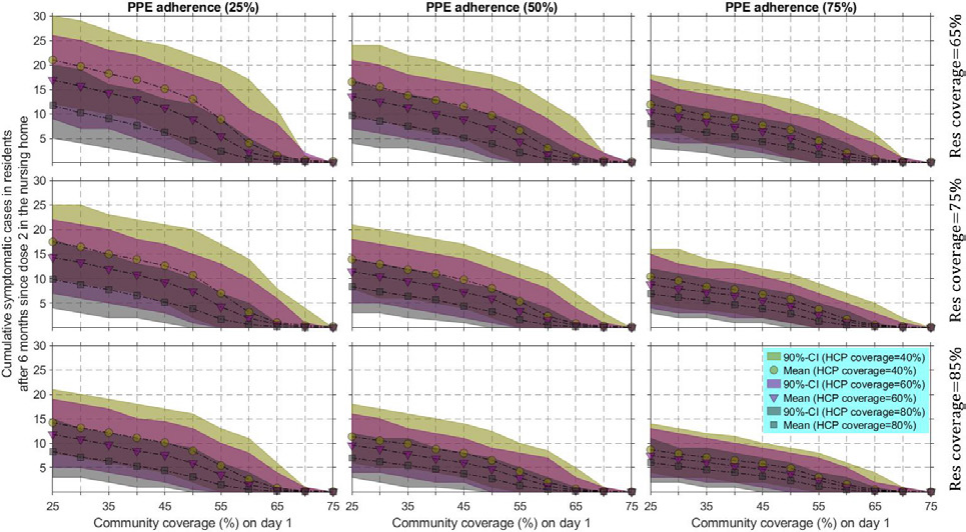

In each panel, we plotted the mean number of cumulative symptomatic cases of COVID-19 in NH residents after 6 months since vaccine dose 2 (given 28 days after dose 1) and their 90% confidence interval (CI) for three healthcare personnel (HCP) coverage scenarios: 40%, 60%, or 80%. Coverage in HCP was independently modeled of community coverage. The top row is for NH resident coverage of 65%, the middle for 75%, and the bottom row for 85%. The columns (left to right) are for facility-level PPE adherence of 25% (low adherence), 50% (intermediate adherence), and 75% (high adherence). Weekly asymptomatic testing of HCP and twice-weekly outbreak testing in the facility were modeled with an assumed point-of-care test sensitivity of 80% (symptomatic persons) and 60% (asymptomatic persons) and with specificity of 100% and test turnaround time of 15 minutes.

Figure 2. Probability of a COVID-19 outbreak in a nursing home (NH) decreased with increase in vaccine coverage in the community or in healthcare personnel (HCP).

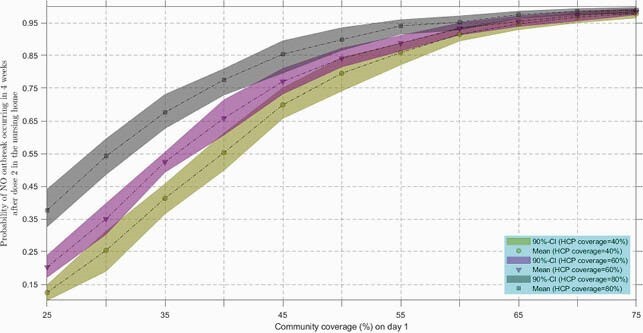

An outbreak is defined as an occurrence of 2 or more cases within 4 weeks of dose 2. Probability of no outbreak was calculated by counting how many simulations out of a total of 1000 simulations had ≤1 symptomatic case in NH residents or HCP within 4 weeks after dose 2 was administered in the nursing home. The first vaccine dose in residents and HCP was assumed to be given on day 1, and the second dose 28 days later. A probability value and its 90%-confidence interval (CI) at a given community and HCP coverage was calculated by pooling model outputs for 9 sets (3 PPE adherence values X 3 resident coverage levels) of model simulations. Simulations were performed assuming no asymptomatic testing or facility-wide outbreak testing.

**Conclusion:**

Results suggest that increasing community vaccination coverage leads to fewer infections in NH residents. Testing asymptomatic residents and staff may have limited value when vaccination coverage is high. High adherence to recommended PPE may increase the likelihood that future COVID-19 outbreaks can be contained.

**Disclosures:**

**John A. Jernigan, MD, MS**, Nothing to disclose

